# Ablation of mitral annular flutter ablation utilizing a left atrial anterior line versus a lateral mitral isthmus line: a systematic review and meta-analysis

**DOI:** 10.1007/s10840-021-00943-x

**Published:** 2021-02-04

**Authors:** Omar M. Aldaas, Florentino Lupercio, Andrew Y. Lin, Frederick T. Han, Kurt S. Hoffmayer, Farshad Raissi, Gordon Ho, David Krummen, Gregory K. Feld, Jonathan C. Hsu

**Affiliations:** 1 Division of Cardiac Electrophysiology, University of California San Diego Health System, 9452 Medical Center Drive, San Diego, La Jolla, CA 92037 USA; 2 Cardiac Electrophysiology Section, Division of Cardiology, Department of Medicine, University of California – San Diego, 9452 Medical Center Drive, 3rd Floor, Room 3E-417, San Diego, La Jolla, San Diego, CA 92037 USA

**Keywords:** Atrial fibrillation, Mitral annular flutter, Catheter ablation, Left atrial anterior wall, Lateral mitral isthmus

## Abstract

**Purpose:**

Mitral annular flutter (MAF) is a common arrhythmia after atrial fibrillation ablation. We sought to compare the efficacy and safety of catheter ablation utilizing either a left atrial anterior wall (LAAW) line or a lateral mitral isthmus (LMI) line.

**Methods:**

We performed a systematic review for all studies that compared LAAW versus LMI lines. Risk ratio (RR) and mean difference (MD) 95% confidence intervals were measured for dichotomous and continuous variables, respectively.

**Results:**

Four studies with a total of 594 patients were included, one of which was a randomized control trial. In the LMI ablation group, 40% of patients required CS ablation. There were no significant differences in bidirectional block (RR 1.26; 95% CI, 0.94–1.69) or ablation time (MD −1.5; 95% CI, −6.11–3.11), but LAAW ablation was associated with longer ablation line length (MD 11.42; 95% CI, 10.69–12.14) and longer LAA activation delay (MD 67.68; 95% CI, 33.47–101.89.14) when compared to LMI. There was no significant difference in pericardial effusions (RR 0.36; 95% CI, 0.39–20.75) between groups and more patients were maintained sinus rhythm (RR 1.19; 95% CI, 1.03–1.37, *p* = 0.02) who underwent LAAW compared to LMI.

**Conclusion:**

Ablation of mitral annular flutter with a LAAW line compared to a LMI line showed no difference in rates of acute bidirectional block, ablation time, or pericardial effusion. However, LAAW ablation required a longer ablation line length, resulted in greater LAA activation delayed and was associated with more sinus rhythm maintenance, with the added advantage of avoiding ablation in the CS.

## Introduction

Mitral annular flutter (MAF) is the most common left atrial macro-reentrant atrial arrhythmia following catheter ablation of atrial fibrillation (AF) [1]. MAF is often resistant to both rate-controlling and antiarrhythmic drugs, thus necessitating catheter ablation for treatment [2]. The two most common approaches for ablation of peri-mitral flutter include a left atrial anterior wall (LAAW) line and a lateral mitral isthmus (LMI) line. While the LAAW line is drawn between the anterior mitral isthmus and right superior pulmonary vein (or occasionally the left superior pulmonary vein or roof line), the LMI line is drawn between the left lower pulmonary vein and LMI. Both approaches have been shown to be effective, but the LMI line often requires additional coronary sinus (CS) ablation to achieve bidirectional block [3–5]. The purpose of our current study was to perform a systematic review of the literature and meta-analysis to compare the efficacy and safety of both approaches.

## Methods

We searched PubMed, clinicaltrials.gov, Medline, Google Scholar, and the Cochrane Central Register of Clinical Trials (Cochrane Library, Issue 09, 2017). This was assessed up to May 2020. No language restriction was applied. The reference list of all eligible studies was also reviewed. Search terms included (*Mitral Annular Flutter* OR *Atrial Fibrillation*) and (*Mitral Isthmus Ablation* or *Anterior Mitral Ablation*) and (*Catheter Ablation*).

Studies were selected by two independent reviewers. The PRISMA statement for reporting systemic reviews and meta-analyses was applied to the methods for this study [6]. The studies had to fulfill the following criteria to be considered in the analysis: (1) Studies had to have compared outcomes in patients who underwent ablation with LAAW versus LMI lesion sets; (2) Studies had to have compared and reported rates of achieving bidirectional block, ablation times, ablation line length, LAA activation delay, rates of pericardial effusions, and/or maintenance of sinus rhythm; (3) Studies must have been published in a peer-reviewed scientific journal.

We aimed to compare the efficacy and safety between LAAW and LMI lines. Two authors (F.L. and O.M.A.) independently performed literature search and extracted data from eligible studies. Outcomes were extracted from original manuscripts and supplementary data. Information was gathered using standardized protocol and reporting forms. Disagreements were resolved by consensus. Two reviewers (F.L. and O.M.A.) independently assessed the quality items and discrepancies were resolved by consensus or involvement of a third reviewer (J.C.H), if necessary.

Two authors (F.L. and O.M.A.) independently assessed the risk of bias of the included trials using standard criteria defined in the Cochrane Handbook for Systematic Reviews of Interventions. Discrepancies were resolved by discussion or adjudication by a third author (J.C.H.).

Data was summarized across treatment arms using the Mantel-Haenszel risk ratio (RR), inverse variance mean difference (MD). The Mantel-Haenszel methods are the fixed-effect methods used when event rates are low or study size is small, as the estimates of the standard errors of the effect estimates that are used in the inverse variance methods may be poor. Heterogeneity of effects was evaluated using the Higgins *I*-squared (*I*^2^) statistic. Random effects models for analyses were used with high heterogeneity (defined as *I*^2^ > 25%); otherwise, fixed effects models of DerSimonian and Laird were used. Funnel plot analysis was used to address publication bias. The statistical analysis was performed by the *Review Manager (RevMan) Version 5.3. Copenhagen: The Nordic Cochrane Centre, The Cochrane Collaboration, 2014*. Descriptive statistics are presented as means and standard deviations (SD) for continuous variables or number of cases (*n*) and percentages (%) for dichotomous and categorical variables.

## Results

### Study selection

**Fig. 1:**
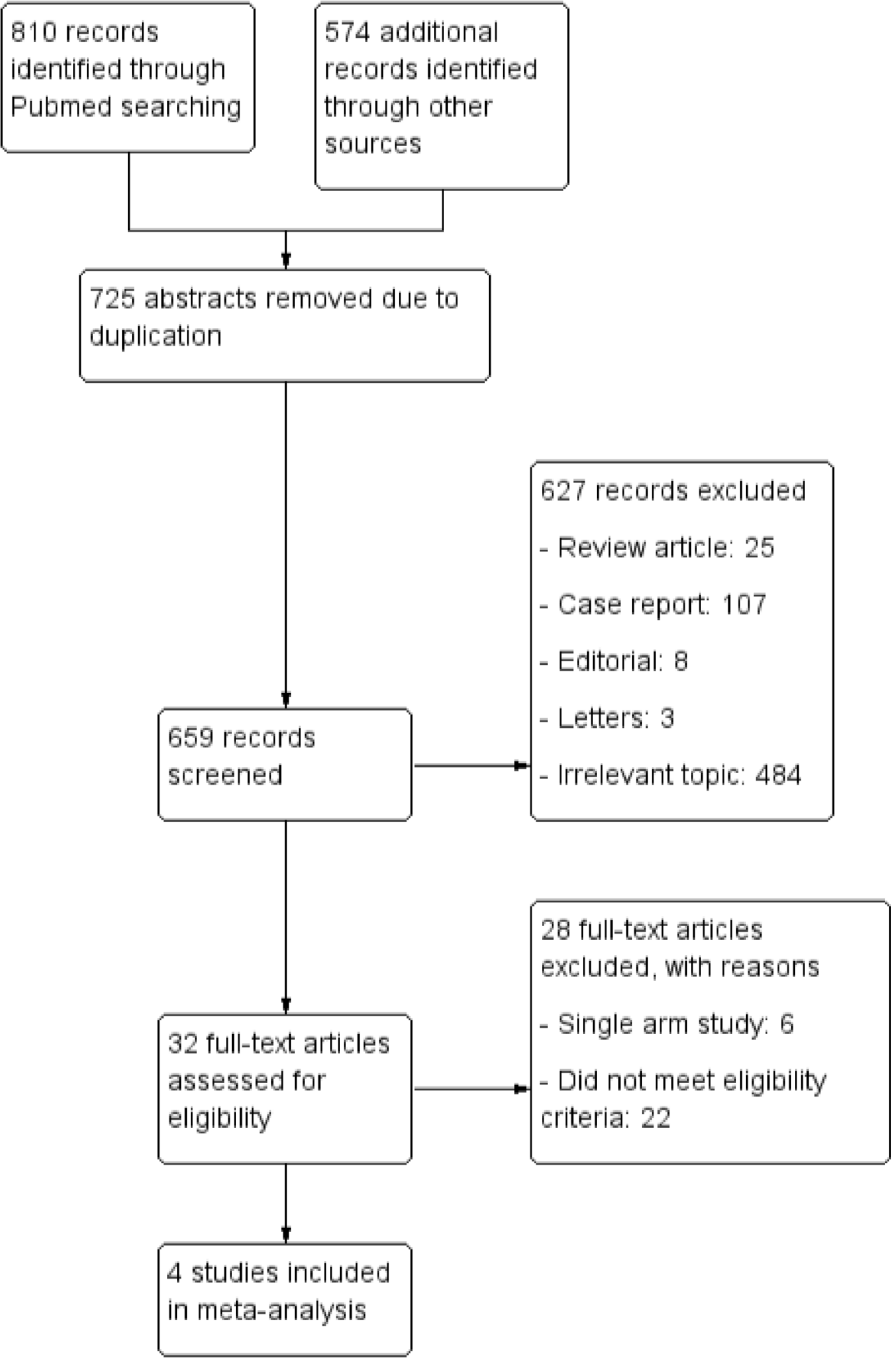
Flow chart showing selection of studies

The initial search resulted in 1384 abstracts, of which 725 were duplications and 627 were excluded based on titles and abstracts (Fig. 1). We included four studies in our final analysis, including one prospective randomized control trial [7], two prospective nonrandomized studies [8, 9], and one retrospective study [10].

### Study characteristics

Baseline demographics of patients included in the four studies are summarized in Table 1. We included a total of 594 patients. Patients were predominately male and many had failed anti-arrhythmic medications. Overall, 318 patients (54%) underwent LAAW ablation and 276 patients (46%) had LMI ablation. Study characteristics are shown in Table 2. A mitral isthmus line was drawn for documented MAF in 25% of patients in the study by Heumer et al. and 93% of patients in the study by Maheshwari et al. [8, 10]. The remainder of patients had a mitral isthmus line drawn empirically [7, 9]. In all studies, patients underwent pulmonary vein isolation (PVI), cavo-tricuspid isthmus ablation, and left atrial (LA) roof line ablation, following varied protocols. LAAW ablation was performed by connecting the right superior pulmonary vein (PV) to the mitral annulus in two studies [7, 10], left superior PV to the anterior mitral annulus in one study [8], and LA roof line to the mitral annulus in one study [9]. LMI was performed by ablating from the left inferior PV to the posterolateral mitral annulus. Two studies reported use of a contact force catheter [9, 10] and steerable sheaths [8, 10], whereas the other two studies did not report whether or not these items were used.

**Table 1:** Patient demographics

Study	Pak et al. [9]		Huemer et al. [8]		Zhang et al. [7]		Maheshwari et al. [10]	
	LAAWL	LMI	LAAWL	LMI	LAAWL	LMI	LAAWL	LMI
Patients – *n*	100	100	40	40	100	100	78	36
Male	76 (76)	83 (83)	28 (70)	23 (58)	54 (54)	58 (58)	45 (58)	26 (72)
Age – yr.	60 ± 11	59 ± 11	66 ± 8	66 ± 9	57 ± 12	59 ± 14	67 ± 11	69 ± 8
BMI – kg/m^2^	NR	NR	28 ± 5	27 ± 5	NR	NR	30 ± 6	29 ± 4
Ejection fraction - %	53 ± 9	56 ± 8	54 ± 7	54 ± 9	56 ± 9	58 ± 9	57 ± 11	55 ± 11
Left atrial size – mm.	44 ± 7	43 ± 7	44 ± 5	45 ± 5	44 ± 7	42 ± 6	NR	NR
Left atrial volume – ml.	179 ± 13	170 ± 12	NR	NR	115 ± 19	122 ± 13	NR	NR
AF duration – yr.	4.6 ± 4.0	4.4 ± 1.5	2.9 ± 3.1	2.4 ± 2.9	3.8 ± 3.7	4.1 ± 3.5	NR	NR
Failed AAD – no.	NR	NR	1.6 ± 0.8	1.6 ± 0.8	2.1 ± 0.8	2.1 ± 1.4	NR	NR
Prior AF ablations	0 (0)	0 (0)	15 (38)*	15 (38)*	NR	NR	60 (77)^†^	28 (78)^‡^
Comorbidities	NR	NR						
HTN CAD DM			32 (80) 3 (8) NR	33 (83) 9 (23) NR	46 24 17	48 26 15	54 (69) 21 (27) 12 (15)	28 (78) 12 (33) 10 (28)

*AAD*, anti-arrhythmic drug; *AF*, atrial fibrillation; *BMI*, body mass index; *CAD*, coronary artery disease; *DM*, diabetes; *HTN*, hypertension; *LAAWL*, left atrial anterior wall line; *LMI*, lateral mitral isthmus line; *NR*, not reported

^*^Pulmonary vein isolation only

^†^18 patients had prior mitral isthmus line ablation

^‡^15 patients had prior mitral isthmus line ablation

**Table 2:** Study characteristics

Study	Pak et al. [9]	Huemer et al. [8]	Zhang et al. [7]	Maheshwari et al. [10]
Study design	Prospective Non-randomized Multi-center	Prospective Non-randomized Single center	Prospective Randomized Single center	Retrospective Non-randomized Single center
Mean follow up – mo.	23.3 ± 7.4	20	31.8 ± 9.4	NR
Type of arrhythmia	Persistent AF	Persistent AF Mitral annulus flutter	Persistent AF	Persistent AF Mitral annular flutter
Ablation strategy	PVI, CTI ablation, LA roof line	PVI and LA roof line	PVI and LA roof line	PVI, CTI ablation if CTI-dependent flutter present
Mapping system	CARTO NavX	NavX	CARTO	CARTO NavX RHYTHMIA
Ablation catheter	Celsius NaviStar	CoolPath Duo	NR	THERMOCOOL ST TERMOCOOL STSF TactiCath INTELLANAV
Contact force	Yes	NR	NR	Yes
Time per lesion	50 s	NR	60 s or until dramatic reduction or elimination of local potential	40 s
Steerable sheath	NR	Yes	NR	Yes
LAAWL	Linear ablation from mitral annulus passing noncoronary cusp of aortic valve to LA roof line.	Left superior PV to anterior mitral annulus in front of the orifice of the LAA.	Linear ablation between anterolateral mitral annulus to the right superior PV ostium, medial to the LAA orifice.	Linear ablation from the anteroseptal mitral annulus to right superior PV. Ablation of Bachmann’s bundle in RA if needed.
LMI	Linear ablation from inferior border of left PVI line to posterolateral mitral annulus. CS ablation if needed to achieve LMI block.	Linear ablation of the shortest distance between left inferior PV to posterolateral mitral annulus. CS ablation if needed to achieve LMI block.	Linear ablation between lateral mitral annulus and the left inferior PV ostium. CS ablation if needed to achieve LMI block.	Linear ablation from lateral mitral annulus to left inferior PV. CS ablation if needed to achieve LMI block.
Monitoring	ECG at 1, 3, 6, 9, 12 months. Holter or event monitor at 3, 6, and 12 months.	ECG at 48 h. Holter monitor at 3, 6, and 12 months or loop recorder implantation.	ECG at 1, 3, 6, 9, and 12 months. Holter monitor at 3, 6, 12, and 24 months.	Two 30-day wearable monitors within 12 months, loop recorder, or device interrogation.

*AF*, atrial fibrillation; *CS*, coronary sinus; *CTI*, cavo-tricuspid isthmus; *ECG*, electrocardiogram; *LA*, left atrium; *LAAWL*, left atrial anterior wall line; *LMI*, lateral mitral isthmus line; *NR*, not reported; *PVI*, pulmonary vein isolation; *RA*, right atrium

### Quality assessment

**Fig. 2:**
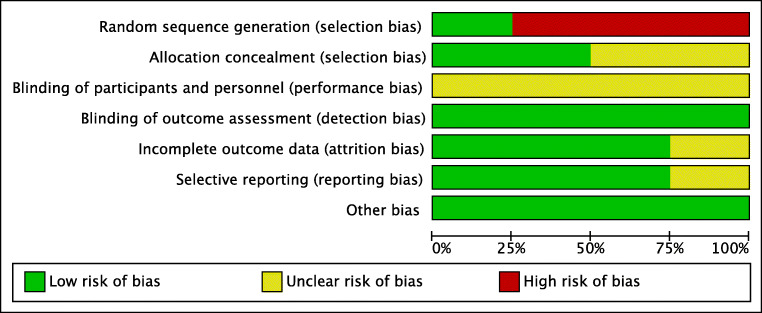
Risk of bias graph. Review authors’ judgements about each risk of bias item presented as percentages across all included studies, according Cochrane Handbook for Systematic Reviews of Interventions

**Fig. 3:**
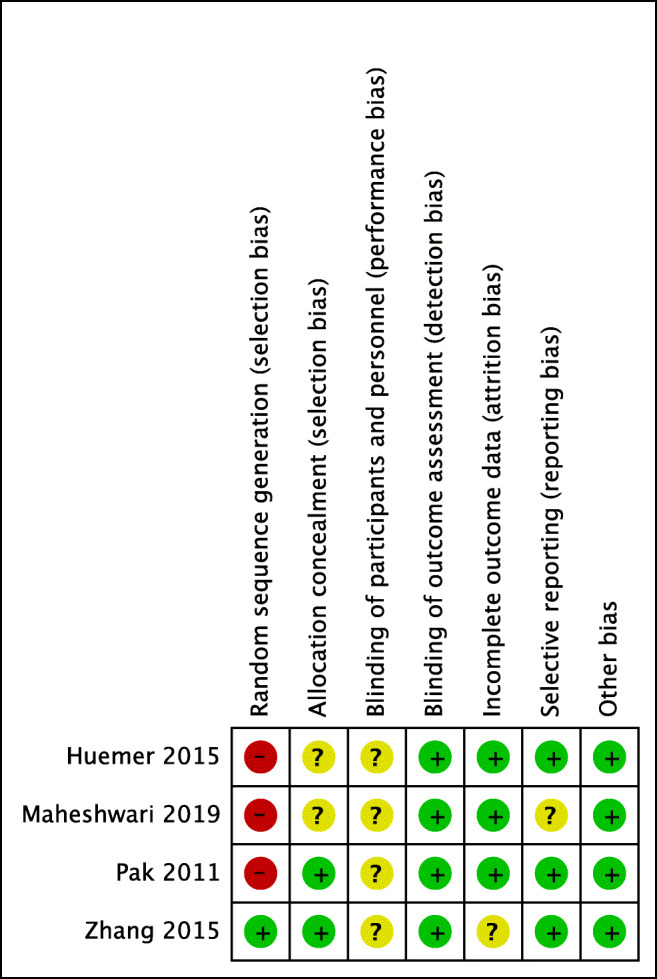
Risk of bias summary. Review authors’ judgements about each risk of bias item for each included study, according Cochrane Handbook for Systematic Reviews of Interventions

The risk of bias is summarized in Figs. 2 and 3. Three studies were considered to be at “high risk” for selection bias due to non-randomization [8–10]. All studies were considered to be at “unclear risk” for performance bias as blinding methodology was not reported. One study was considered to have “unclear risk” for attrition bias given the data on attrition after randomization of cohorts is unavailable [7].

### Study endpoints

**Fig. 4:**
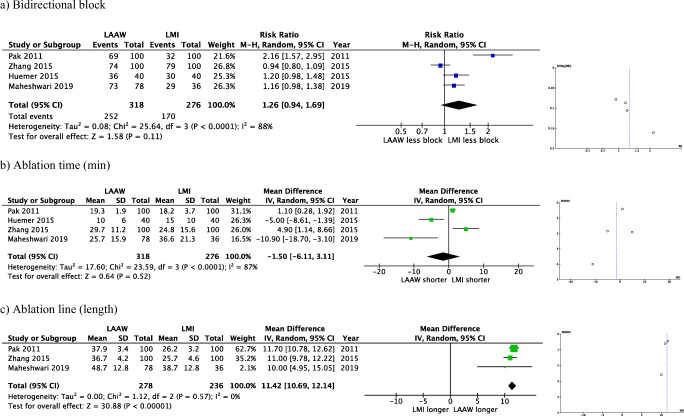
Forrest plots of the comparative analysis of efficacy outcomes in patients with left atrial anterior wall line versus lateral mitral isthmus line. **a** Bidirectional block. **b** Ablation time. **c** Ablation line length

**Fig. 5:**
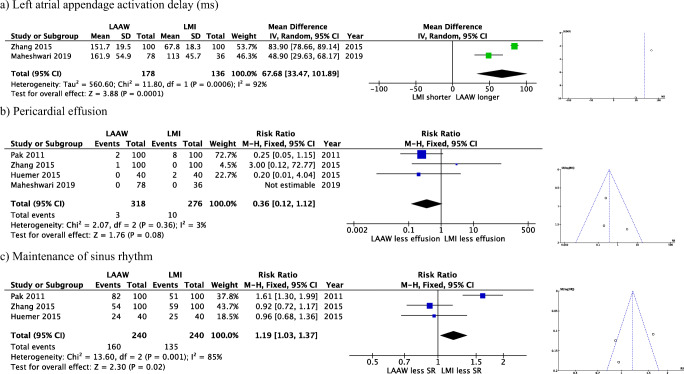
Forrest plots of the comparative analysis of procedural outcomes in patients with left atrial anterior wall line versus lateral mitral isthmus line. **a** Left atrial appendage delay measured in mm. **b** Pericardial effusion. **c** Maintenance of sinus rhythm

Study endpoints between the LAAW and LMI groups are summarized in Figs. 4 and 5. There were no significant differences in achievement of bidirectional block (RR 1.26; 95% CI, 0.94–1.69, *p* = 0.11) between LAAW (79%) and LMI (62%) groups. Of the 170 patients who achieved bidirectional block with a LMI line, 107 required additional ablation in the coronary sinus. One study proceeded with the alternative ablation strategy when the initial attempt failed to achieve conduction block (i.e., 5 patients who failed to achieve conduction block in the LAAW group underwent LMI ablation of which 4 were successful, and of 7 patients in the LMI group who failed to achieve conduction block, 3 underwent LAAW ablation but none were successful) [10]. Although there was no difference in ablation time in LAAW and LMI groups (20.6 ± 7.7 versus 22.1 ± 11.4 min) (MD −1.5; 95% CI, −6.11–3.11, *p* = 0.52), LAAW ablation was associated with longer ablation line length (37.7 ± 3.9 versus 26.3 ± 3.9 mm) (MD 11.42; 95% CI, 10.69–12.14, *p* < 0.01). LAAW ablation was associated with significant LAA activation delay (156.4 ± 35.9 versus 88.7 ± 31.0 ms) (MD 67.68; 95% CI, 33.47–101.89, *p* < 0.01), a trend toward fewer pericardial effusions (0.9 versus 3.6%) (RR 0.36; 95% CI, 0.12–1.12, *p* = 0.08), and a higher proportion of patients maintained in sinus rhythm at follow-up (66.6 versus 56.3%) (RR 1.19; 95% CI, 1.03–1.37, *p* = 0.02).

## Discussion

This is the first systematic review and meta-analysis of studies comparing procedural and peri-procedural outcomes between LAAW and LMI ablation lines for treatment of MAF. The results of this meta-analysis show that there are no significant differences in rates of bidirectional block, ablation time, and risk of pericardial effusion between ablation approaches. However, LAAW ablation necessitates a longer ablation line length and results in delayed LAA activation, while improving maintenance of sinus rhythm during follow-up and foregoing the need for ablation of the CS.

While there are two approaches to mitral isthmus line ablations, the LMI line was first described and has been the conventional approach [4]. However, ablation using this line often necessitates ablation within the CS, which may result in complications such as coronary spasm or occlusion, perforation of the CS, and pericardial tamponade [3, 11–13]. Furthermore, achieving conduction block with an LMI line may be challenging given the increased myocardial thickness, convective cooling from the CS and left circumflex artery, and epicardial connections [14, 15]. A superolateral mitral isthmus line, where the left-sided pulmonary veins are connected with the mitral annulus along the posterior base of the left atrial appendage, may be a potential alternative. This method targets the mitral isthmus at its thinnest portion where bridging of an endocardial linear lesion by muscular sleeves encircling the CS is unlikely. While a high acute success rate of bidirectional block using endocardial ablation only has been demonstrated (98.2 versus 87.7% with the traditional LMI line, *p* = 0.06), it may be associated with a higher incidence of pericardial tamponade (5.2 versus 0% with the traditional LMI line, *p* = 0.24) [16].

The LAAW line was described subsequently in an attempt to overcome these limitations. Conduction block may be easier to achieve in the low-voltage area of the LAAW; the rigid structure of the posterior wall of the aorta facilitates good contact pressure; ablation across the septo-atrial bundle reduces left atrial critical mass and may block multi-loop reentry around the mitral valve annulus. In addition, complex fractionated atrial electrograms are usually localized to the antero-septum or base of the left atrial appendage and may be disrupted by a LAAW ablation line [17–19], Furthermore, although the LAAW line is longer, it encounters less endocardial obstacles such as diverticula and pouches, which means that it usually requires a lower amount of radiofrequency energy to create a transmural lesion [20, 21]. However, LAAW line ablation has its own limitations. LAAW ablation results in significant conduction delay to the left atrial appendage and may result in intra-atrial or atrio-ventricular dyssynchrony [8, 9]. While cardiac MRI follow-up of 29 patients did not show significant hemodynamic derangements, there is still lack of data on the possible consequences on left atrial appendage flow velocity [9]. Inadvertent disconnection of the left atrial appendage may result from disruption of Bachmann’s bundle and has led to a concern about exposing the patient to a higher risk of thrombus formation and systemic embolization (e.g., stroke) [7, 22, 23], but evidence thus far has been conflicting [7, 24]. Furthermore, failure to achieve durable conduction block, which occurs in a significant percentage of patients [3, 9, 25–28], can result in areas of slow conduction along the line that can become substrate for macroreentry [29, 30]. Difficulties in attaining complete conduction block at the mitral isthmus have been attributed to a lack of contact pressure and catheter stability, myocardial thickness, and possible epicardial sleeves at the CS [26, 28, 31]. There is also the potential to injure the sinus nodal artery that runs along the left atrial roof which can result in sinus node dysfunction [32].

While this study did not show any difference in the safety of LAAW and LMI lines immediately post-procedure, long-term follow-up is lacking. As previously mentioned, the stroke risk associated with conduction delay from a LAAW ablation is not known. Matsuo et al. looked at 50 patients over an average follow-up of 19 ± 4 months undergoing ablation of AF that had MAF which was present during AF ablation (24/50) or during follow-up (26/50). The incidence of MAF during the index ablation was significantly higher in patients who required ablation of the mitral isthmus as part of the stepwise approach to terminate persistent AF than in those who did not (23 (9/39) versus 8% (5/59), *p* = 0.04). Following the procedure, MAF was more frequent in patients with prior MI ablation than in those without (41 versus 15%, *p* = 0.01) [25]. Anousheh et al. showed that the risk of MAF was four times higher if block was not achieved during the first procedure. Although this study had an acute rate of mitral isthmus line block of 83%, four of seven patients had MAF at an average follow-up of 18 ± 5 months due to recovery of conduction across the MI line [33]. Wong et al. found resumption of conduction across the mitral isthmus line to be present in 44% of redo procedures, with long-term maintenance of bidirectional block in 58% of patients who underwent a repeat ablation. After a mean follow-up of 20 ± 9 months, 73% of patients remained free from atrial flutter or tachycardia [12]. Taken together, these data show a large minority of patients develop MAF after a mitral isthmus line when followed out to as far as 20 months.

## Study limitations

This study has several important limitations that should be acknowledged. First, the studies included in the meta-analysis enrolled heterogeneous populations with variations in study design and ablation protocols, which may limit the generalizability of the results. Second, the decision to perform LAAW or LMI ablation was not standardized but rather based on proceduralist preference in three of the studies. Third, normal values for pre-ablation LAA activation delay were not always defined and it is thus unknown how the effects of endogenous scarring and prior pulmonary vein isolation may play a role in choice of LAAW or LMI lines. Fourth, there was notable heterogeneity in the use of ECGs, Holter monitors, event monitors, loop recorders, or device interrogation at various time intervals, which could have resulted in differential assessment of arrhythmia recurrence rates among studies. Despite these limitations, our study represents the first meta-analysis comparing LAAW and LMI and offers valuable data on the outcomes of these two ablation techniques.

## Conclusion

Ablation of MAF with LAAW line compared to a LMI line showed no difference in rates of bidirectional block, ablation time, and pericardial effusion. However, LAAW ablation necessitates longer ablation line length and results in delayed LAA activation, while improving maintenance of sinus rhythm in follow-up and foregoing the need for ablation of the CS. These findings suggest that the technique used for MAF ablation should be individualized based on operator experience, patient anatomy, and substrate location.

## Abbreviations

CI: Confidence interval
CS: Coronary sinus
LAA: Left atrial appendage
LAAW: Left atrial anterior wall
LMI: Lateral mitral isthmus
MAF: Mitral annular flutter
MD: Mean difference
RR: Risk ratio
